# Supplementary material for: Convergence of orphan quality control pathways at a ubiquitin chain-elongating ligase
Source: Mol Cell. Author manuscript; Available in PMC 2025 Jun 24. (PMC7617804; doi:10.1016/j.molcel.2025.01.002)
Supplement: Document S1. Figures S1–S9 [file EMS206527-supplement-Document_S1__Figures_S1_S9_.pdf]

Molecular Cell, Volume *85*

Supplemental information

Convergence of orphan quality control pathways at a ubiquitin chain-elongating ligase

Sara Carrillo Roas, Yuichi Yagita, Paul Murphy, Robert Kurzbauer, Tim Clausen, Eszter Zavodszky, and Ramanujan S. Hegde

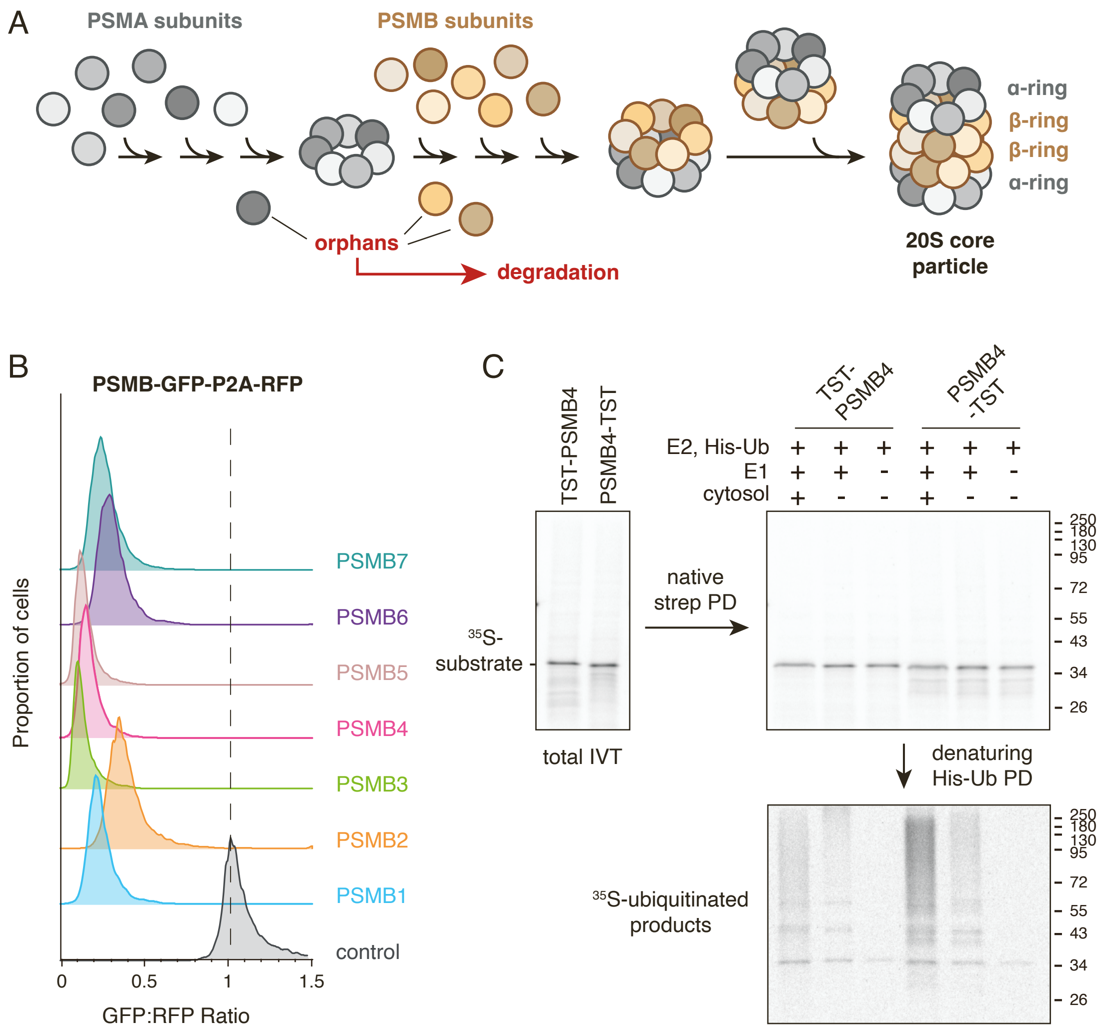

Figure S1. In cell and in vitro analysis of orphaned PSMB subunits, related to Figure 1. (A) Simplified schematic depicting 20S core proteasome assembly and quality control. The hetero-heptameric alpha ring is formed from subunits PSMA1-PSMA7, and the hetero-heptameric beta ring is formed from subunits PSMB1-PSMB7. The 20S core complex is comprised of two rings of each type. Any unassembled subunits (e.g., from imbalanced synthesis), defined as orphans, are degraded. Chaperones, assembly factors and processing steps are not depicted for simplicity. **(B)** Stable-inducible cell lines containing each of the seven PSMBx-GFP reporters and a control GFP reporter (lacking the PSMB insert) were induced with dox for 48h and analyzed by flow cytometry. The stability of each reporter was assessed relative to a co-expressed RFP control (see Fig. 1A), with the GFP:RFP ratio being plotted as a histogram. Note that all of the PSMBx reporters are degraded relative to the control. **(C)** PSMB4 tagged with a N-terminal or C-terminal TwinStrep tag (TST) was translated in rabbit reticulocyte lysate (RRL) with ^{35}S-methione and affinity-purified via TST under native conditions. The affinity-purified products were divided into three aliquots and incubated with E1, E2 (UBE2D1), His-Ub, ATP and cytosol (RRL), as indicated, then subjected to a pulldown via His-Ub under denaturing conditions. Aliquots of the samples at each step of the process were analyzed by SDS-PAGE and autoradiography: total IVT, the total products of the ubiquitination reaction, and the products recovered by His-Ub pulldown.

A

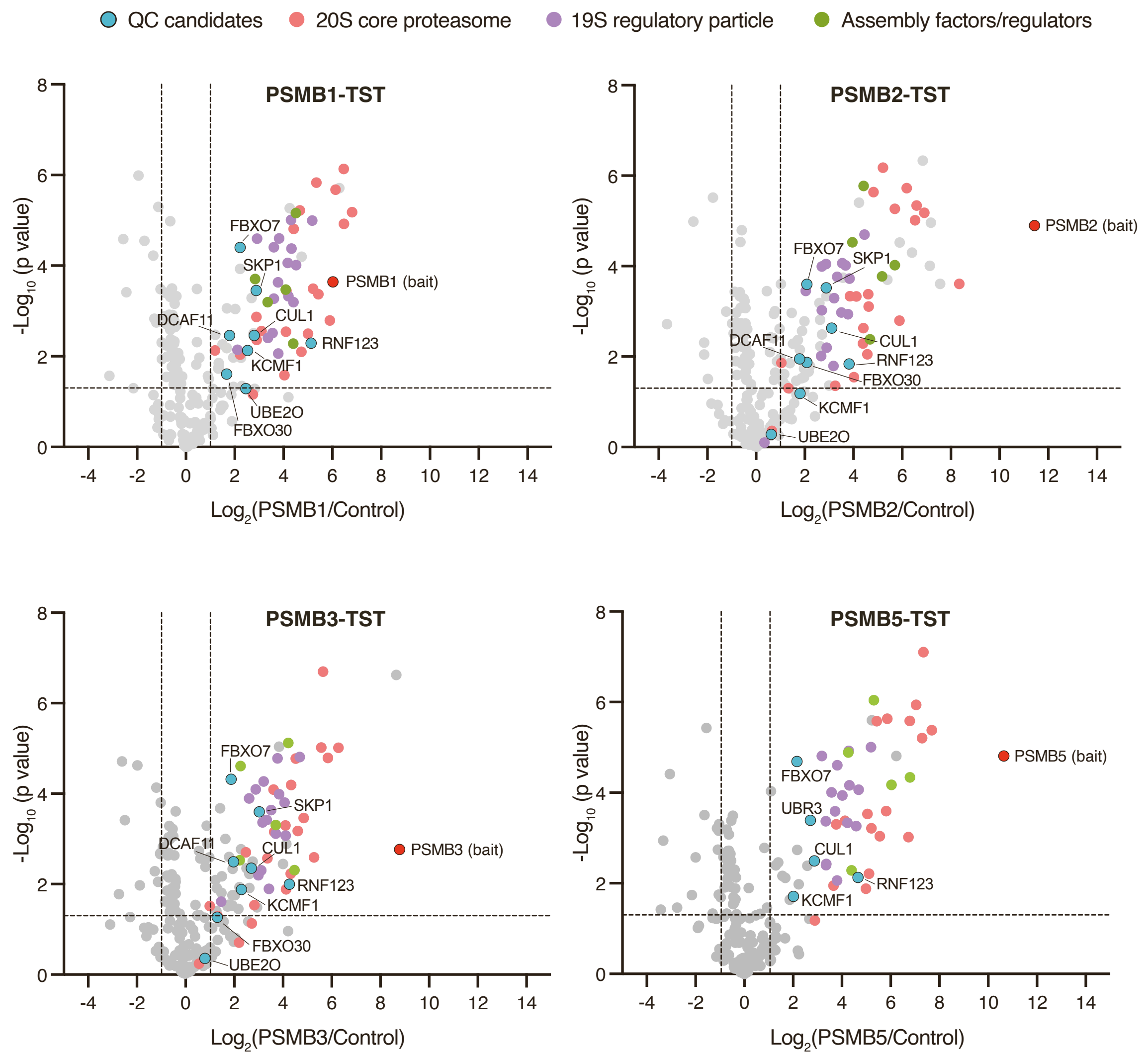

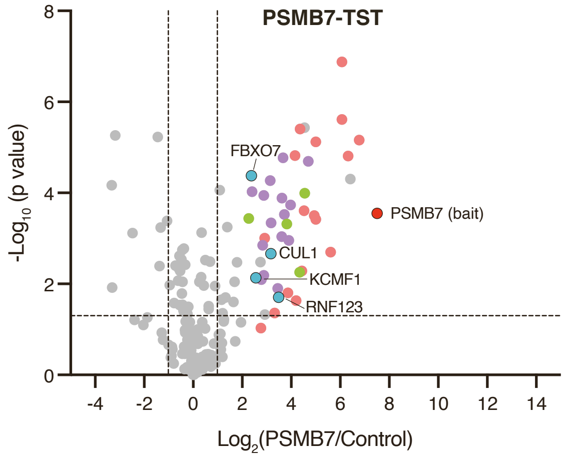

B

	Factor:	enriched with:
QC ligase Candidates	KCMF1	all PSMBs
	RNF123	all PSMBs
	FBXO7	all PSMBs
	CUL1	all PSMBs
	SKP1	PSMB1,2,3,4
	DCAF11	PSMB1,2,3
	FBXO30	PSMB1,2
	UBE2O	PSMB1 only
	UBR3	PSMB5 only
Assembly factors	PSMG1	all PSMBs
	PSMG2	all PSMBs
	POMP	all ex. PSMB7

Figure S2. Identification of quality control candidates of orphaned PSMB subunits, related to Figure 2. **(A)** C-terminal TST-tagged PSMB subunits were translated in RRL, affinity-purified under native conditions and analyzed by label free quantitative mass spectrometry. The volcano plots show proteins enriched in each of the PSMB subunit pulldowns compared to a mock translation which served as a negative control. P-values were calculated by two-sided Student's *t*-test with Benjamini Hochberg correction for multiple comparisons. The translation reaction for PSMB6 failed in this experiment, so results for this sample are not shown. **(B)** Table showing QC ligase candidates and assembly factors that were enriched in at least one of the PSMB pulldowns. Recovery of the assembly factors indicates that a proportion of each translation product begins but does not complete assembly, indicative of a population of orphaned subunits in the reaction.

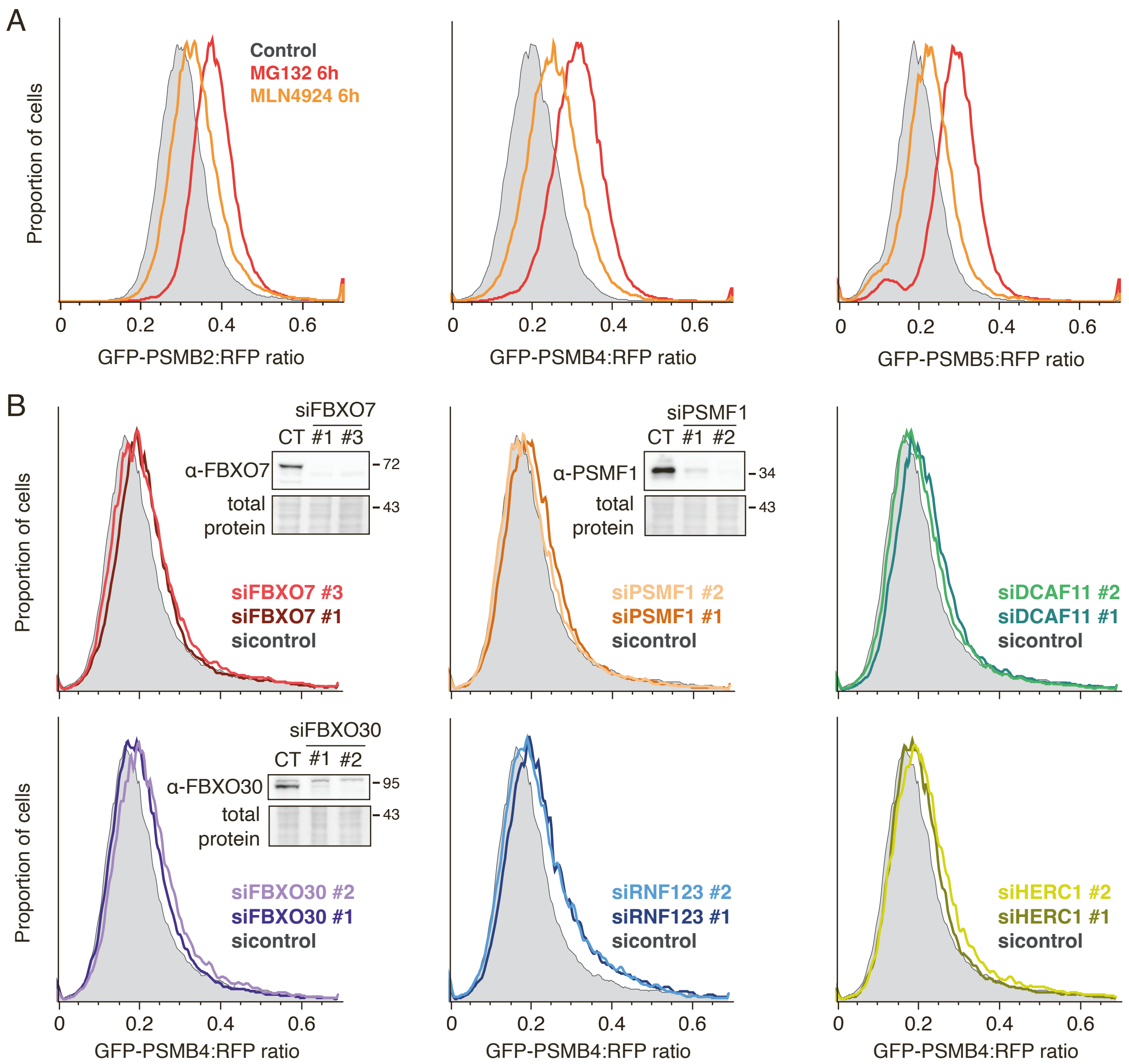

Figure S3. Analysis of quality control candidates in the degradation of orphaned PSMB subunits, related to Figure 2. (A) Stable-inducible cell lines containing the indicated GFP-PSMB reporter were induced with dox for 18h. After removal of dox, cells were treated with 20 µM MG132 or 1 µM MLN4924 (a neddylation inhibitor) for 6 hours, then analyzed by flow cytometry. **(B)** The stable-inducible cell line containing the GFP-PSMB4 reporter was transfected with non-targeting control or the specified siRNAs for a total of 72h. GFP-PSMB4 reporter expression was induced with dox for the last 18h. Cells were then analyzed by flow cytometry and immunoblot (where suitable antibodies were available).

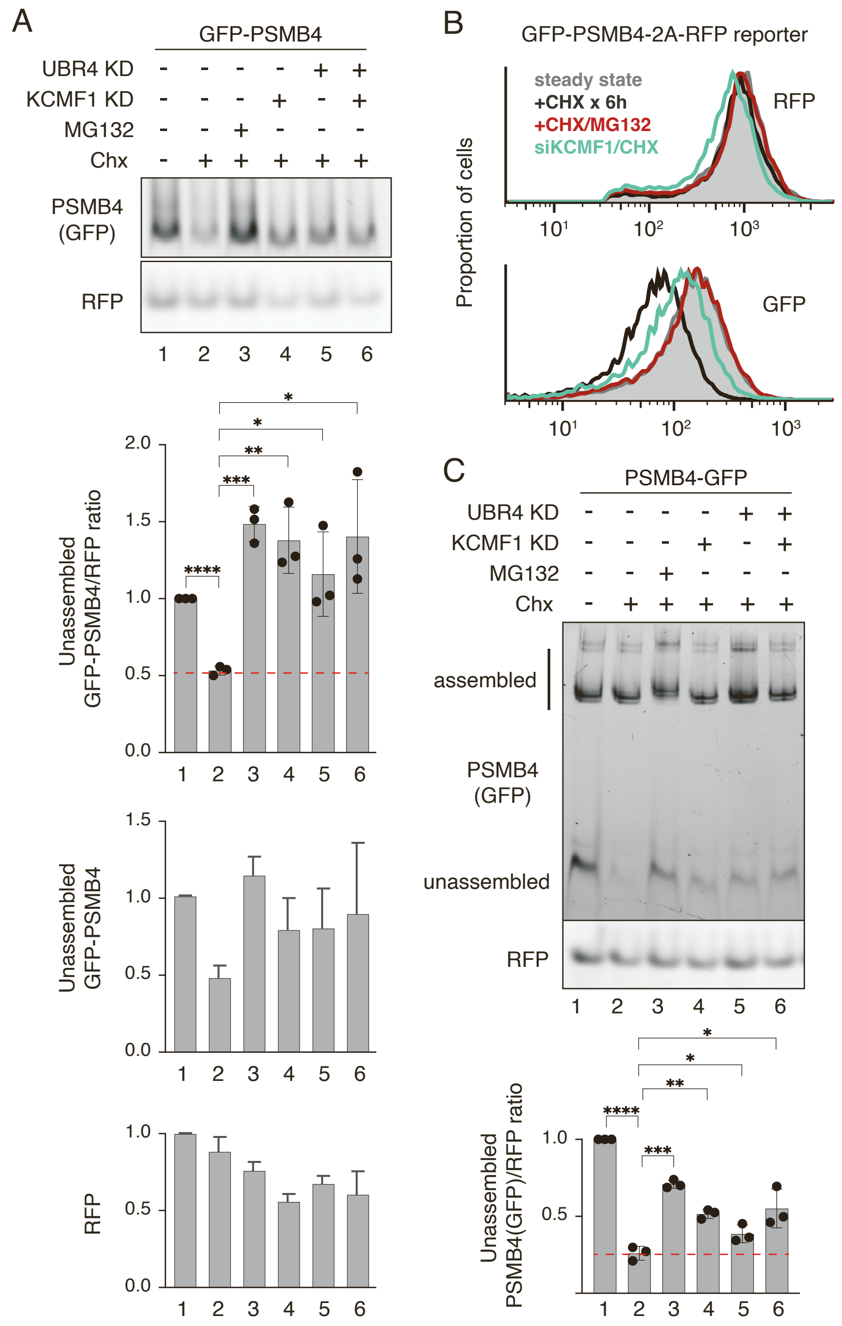

Figure S4. The UBR4-KCMF1 complex is required for degradation of orphaned PSMB4 reporters, related to Figure 2. (A) The GFP-PSMB4 reporter cell line was transfected with non-targeting control, KCMF1 or UBR4 siRNAs for a total of 72h. GFP-PSMB4 reporter expression was induced with dox after 54h for a period of 18h. After removal of dox, cells were treated with 100 µg/mL CHX and 20 µM MG132 for 6h, as indicated. Cell lysates were then analyzed by Native PAGE and in-gel fluorescence to detect GFP and RFP (top). The ratio of unassembled PSMB4-GFP fluorescence to RFP, relative to untreated control, was quantified from three replicates from two independent experiments and plotted (right). Single, double, triple and quadruple asterisk indicate Student's t-test p values less than 0.05, 0.01, 0.001 and 0.0001, respectively. Error bars indicate standard deviation. The individual GFP and RFP signals are shown in separate plots below the ratio plot. Note that knockdown of UBR4 or KCMF1 results in somewhat lower rates of reporter translation (based on RFP levels), possibly due to stress pathway activation. **(B)** The experiment from Fig. 2C shown as individual histograms for GFP-PSMB4 and the RFP control. As seen in the native gels (panel A), KCMF1 knockdown results in slightly reduced reporter translation as judged by less RFP signal. **(C)** Analysis as in panel A but using the PSMB4-GFP reporter cell line. Unlike the N-terminally tagged GFP-PSMB4, the C-terminally tagged PSMB4-GFP reporter can assemble into proteasome complexes (see Fig. 1D). Nonetheless, efficient degradation of the unassembled population of PSMB4-GFP is dependent on KCMF1 and UBR4. The degradation is not as complete as GFP-PSMB4, presumably because a subpopulation of unassembled PSMB4-GFP assembles into proteasome complexes.

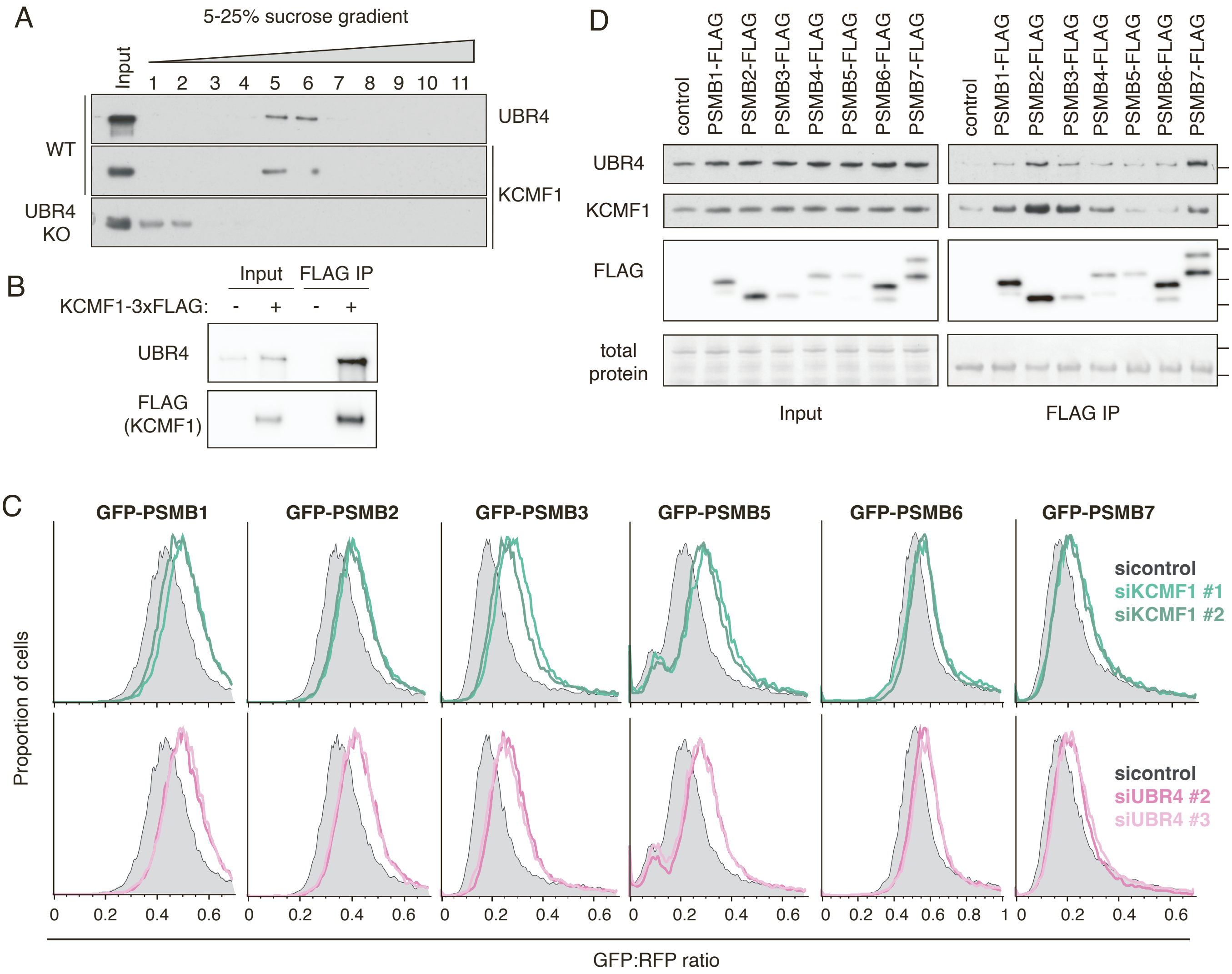

Figure S5. The UBR4-KCMF1 complex is required for the degradation of multiple orphaned PSMB subunits, related to Figure 2. (A) Total cell lysates (Input) from wildtype (WT) or ΔUBR4 cells were separated on a 5-25% sucrose gradient and analyzed by immunoblot. Note that UBR4 and KCMF1 co-migrated as a large complex in fractions 5-6 in WT cells, but that KCMF1 shifted to fractions 1-2 when UBR4 was knocked out. **(B)** ΔKCMF1 cells stably integrated with dox-inducible KCMF1-3xFLAG were induced with dox for 18h and subjected to anti-FLAG IP under native conditions. Input and FLAG IP samples were analyzed by immunoblot. **(C)** Cells stably integrated with inducible GFP-PSMBx reporters were transfected with non-targeting control, KCMF1 or UBR4 siRNAs for a total of 72h. Expression of GFP-PSMBs was induced with dox for the last 18h and cells were analyzed by flow cytometry. **(D)** HEK293T cells transiently transfected with C-terminally FLAG-tagged PSMB subunits were subjected to anti-FLAG IP under native conditions. Input and FLAG IP samples were analyzed by immunoblot.

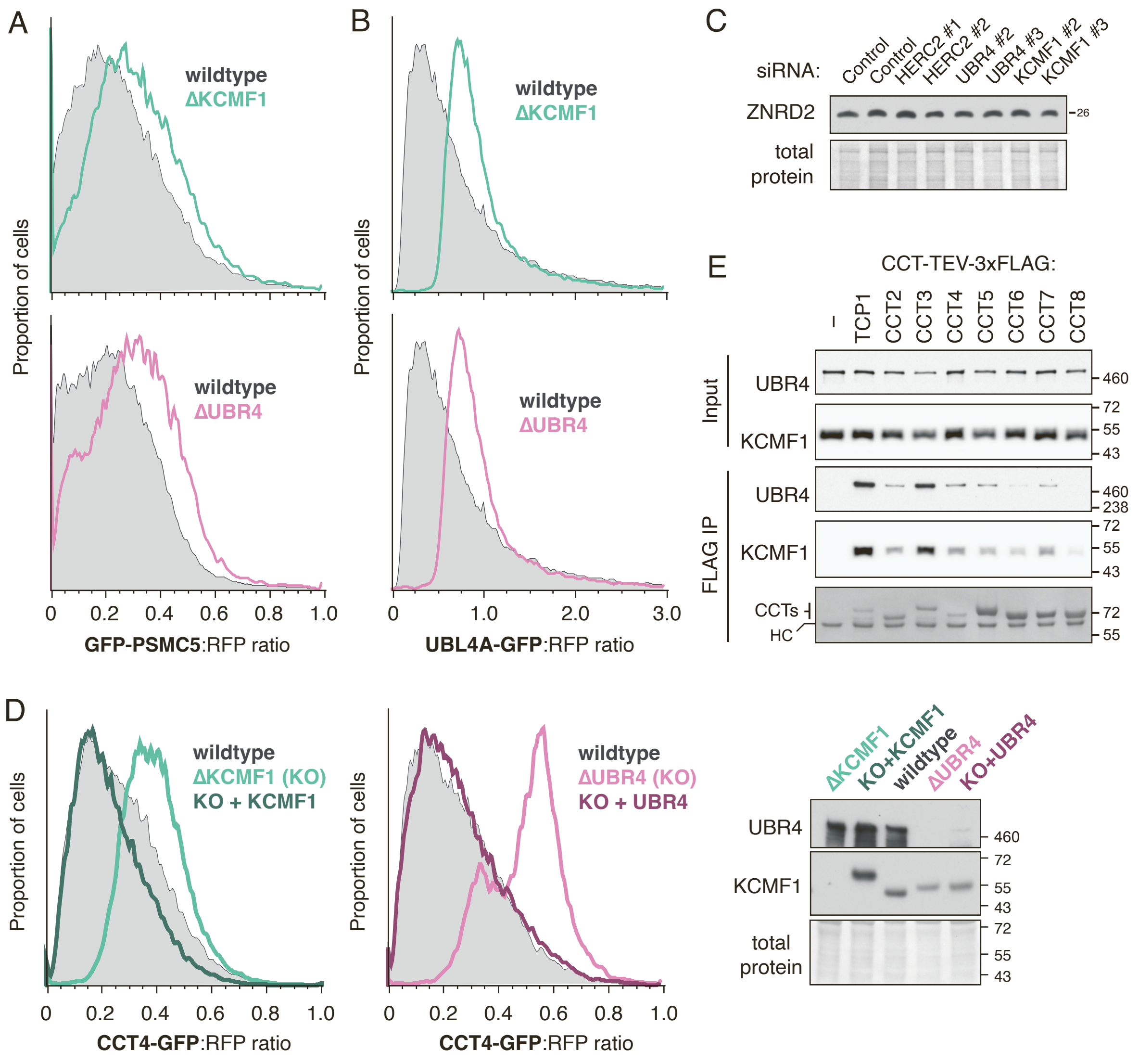

Figure S6. The UBR4-KCMF1 complex is required for the degradation of orphaned PSMC5, UBL4A and CCT4, related to Figure 3. (A) Wildtype, ΔKCMF1 or ΔUBR4 cells were transiently transfected with GFP-PSMC5 reporter for 48h and analyzed by flow cytometry. Note that the steady-state level of orphaned GFP-PSMC5 is higher (relative to the internal RFP control) in both KO cells. **(B)** Same as A but with the UBL4A-GFP reporter. **(C)** Lysates from cells treated with the indicated siRNAs (see Fig. 3A) were analyzed by immunoblot for ZNRD2, whose levels do not change in any of the conditions. **(D)** Wildtype, ΔKCMF1 or ΔUBR4 cells were transiently co-transfected with the CCT4-GFP reporter and KCMF1-3xFLAG or UBR4-3xFLAG constructs, as indicated, for 48h. Cells were then analyzed by flow cytometry (left and middle) or immunoblot (right). By comparison to KCMF1, re-expression of UBR4 is relatively low due to reduced overall transfection efficiency (in terms of both copy number and percent of cells) due to the very large plasmid size. Nonetheless, analysis of the transfected cells by flow cytometry indicates complete rescue of the degradation phenotype. **(E)** HEK293T cells transiently transfected with C-terminally FLAG-tagged CCT subunits were subjected to anti-FLAG IP under native conditions. Input and FLAG IP samples were analyzed by immunoblot for UBR4 and KCMF1, or total protein stain for CCTs.

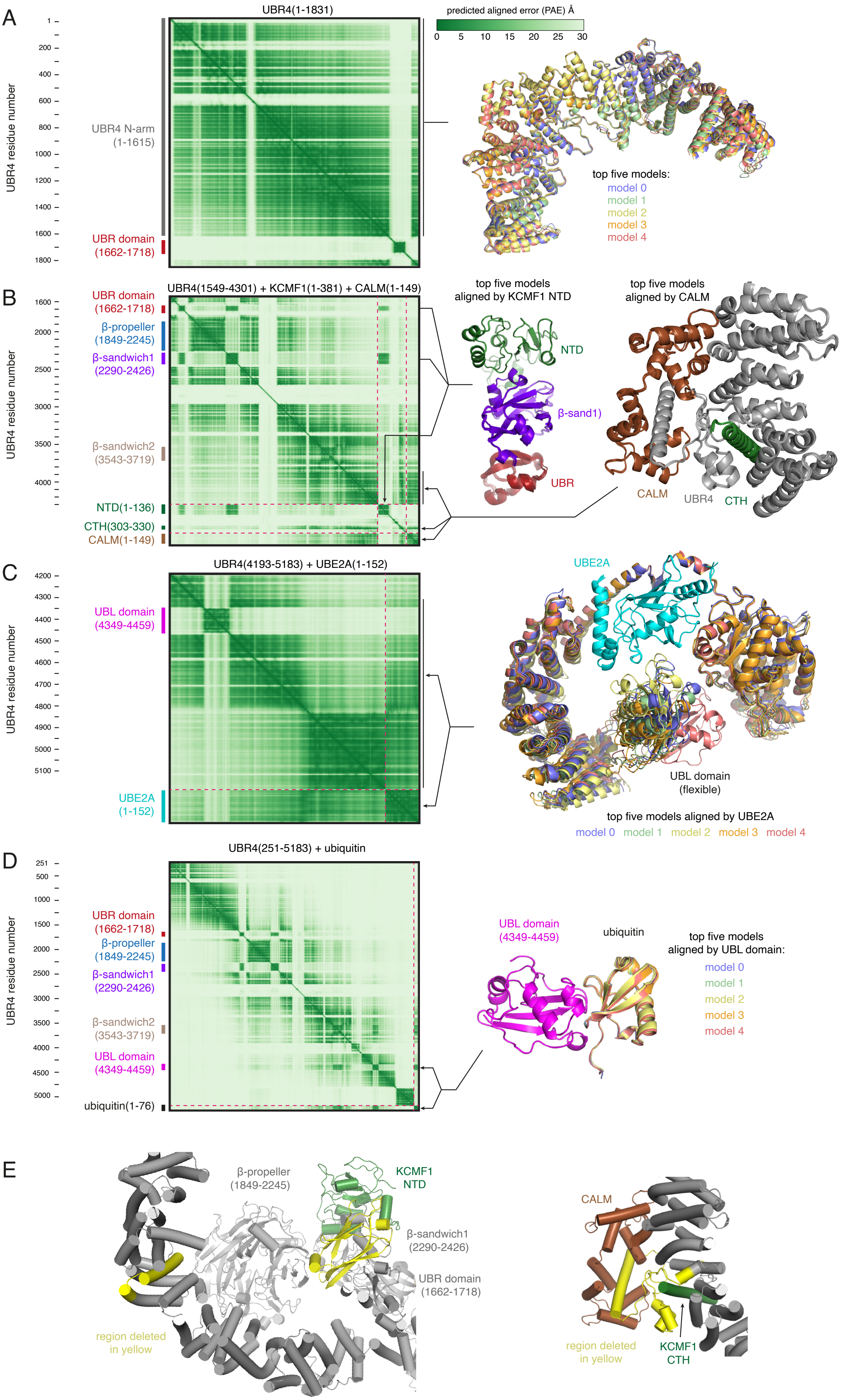
A
UBR4(1-1831)
predicted aligned error (PAE) Å
0 5 10 15 20 25 30
UBR4 residue number
UBR4 N-arm
(1-1615)
UBR domain
(1662-1718)
top five models:
model 0
model 1
model 2
model 3
model 4
B
UBR4(1549-4301) + KCMF1(1-381) + CALM(1-149)
UBR domain
(1662-1718)
β-propeller
(1849-2245)
β-sandwich1
(2290-2426)
β-sandwich2
(3543-3719)
NTD(1-136)
CTH(303-330)
CALM(1-149)
top five models
aligned by KCMF1 NTD
NTD
β-sand1)
UBR
top five models
aligned by CALM
CALM
UBR4
CTH
C
UBR4(4193-5183) + UBE2A(1-152)
UBL domain
(4349-4459)
UBE2A
(1-152)
UBE2A
UBL domain
(flexible)
top five models aligned by UBE2A
model 0 model 1 model 2 model 3 model 4
D
UBR4(251-5183) + ubiquitin
UBR domain
(1662-1718)
β-propeller
(1849-2245)
β-sandwich1
(2290-2426)
β-sandwich2
(3543-3719)
UBL domain
(4349-4459)
ubiquitin(1-76)
UBL domain
(4349-4459)
ubiquitin
top five models
aligned by UBL domain:
model 0
model 1
model 2
model 3
model 4
E
β-propeller
(1849-2245)
KCMF1
NTD
β-sandwich1
(2290-2426)
UBR domain
(1662-1718)
region deleted
in yellow
CALM
region deleted
in yellow
KCMF1
CTH

Figure S7. Structural modelling of the UBR4-KCMF1-UBE2A-CALM complex and its interactions with ubiquitin, related to Figure 5. (A) AlphaFold3 prediction of UBR4(1-1831). Shown at left is the matrix of predicted aligned error for the top predicted model, annotated on the left side with amino acid numbers and key domains. Shown on the right is an alignment of the N-arm for the top five models. The UBR domain, whose fold is predicted with high confidence, is positioned differently in all the models due to its lack of interaction with the N-arm (evident on the PAE plot). Instead, the UBR domain is tethered by two flexible linkers. **(B)** AlphaFold3 prediction of UBR4(1549-4301) with KCMF1 and CALM. The PAE plot is displayed as in A, with the different proteins and domains highlighted on the left. On the right, alignments of the top five models for two key high-confidence interactions are displayed, with arrows indicating the regions of low PAE between the interacting domains. The KCMF1(NTD)-   -sandwich1-UBR models were aligned on the NTD; the CALM-UBR4-CTH models were aligned on CALM. **(C)** AlphaFold3 prediction of UBR4(4193-5183) with UBE2A displayed as in A. Alignment of the top five models was on UBE2A. Note the apparent flexibility of the UBL domain's position due to its attached flexible linkers. **(D)** AlphaFold3 prediction of UBR4 (251-5183) and ubiquitin. Note the high-confidence interaction, indicated on the PAE plot, between the UBL domain of UBR4 and ubiquitin. Shown on the right is an alignment of the top five models (aligned on the UBL domain) of this UBL-ubiquitin module. **(E)** Close-up views of the AlphaFold3-predicted UBR4-KCMF1-CALM composite model highlighting the regions in UBR4 (yellow) that had been previously deleted[35] to created UBR4 mutants deficient in KCMF1 binding (left) or CALM binding (right). Note that although these deletions would disrupt binding to the interacting domain as intended, both of them also encroach on key structural elements of the core UBR4 scaffold.

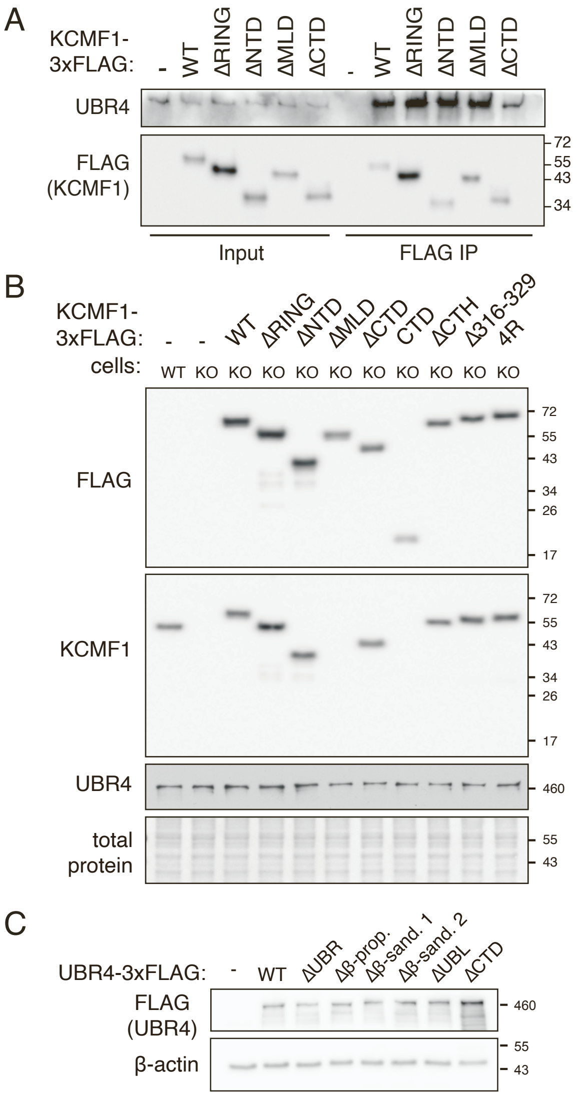

Figure S8. Analysis of KCMF1 deletion mutants, related to Figure 6. (A) ΔKCMF1 cells were transiently transfected with the indicated KCMF1-3xFLAG constructs for 48h. Cells were then subjected to anti-FLAG IP under native conditions, and input and IP samples were analyzed by immunoblot. **(B)** The KCMF1-3xFLAG constructs used in Fig 6A and B were transiently transfected into the indicated cells and the expression levels of KCMF1 and UBR4 were analyzed by immunoblot. Note that the immunogen for the KCMF1 antibody (aa 206-296) is not present in the MLD and CTD constructs. **(C)** Expression of UBR4 mutants used in Fig 6C was analyzed by immunoblot.

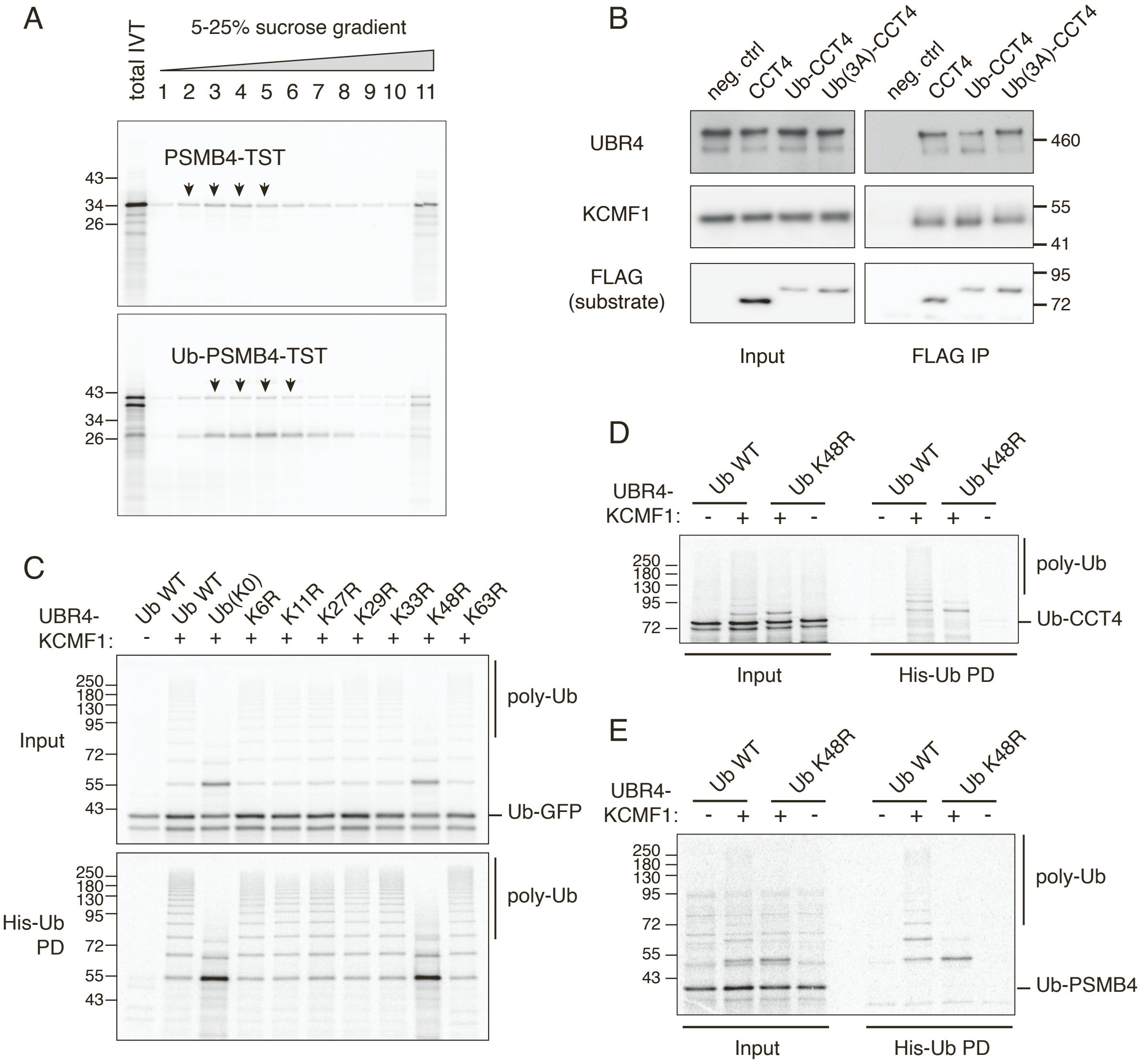

Figure S9. Functional and interaction analysis of the UBR4-KCMF1 complex, related to Figure 7. (A) Preparation of ^{35}S-methionine-labelled PSMB4 and ubiquitin(G76V)-fused PSMB4 using the PURE translation system. After in vitro translation, the reactions were separated on a 5-25% sucrose gradient, and resulting fractions were analyzed by SDS-PAGE and autoradiography. The arrowheads indicate the soluble fractions that were pooled and used as substrates for subsequent *in vitro* ubiquitination assays. Other ubiquitination substrates were prepared similarly. Further analysis revealed that the prominent smaller molecular weight band seen in the Ub-fused translation reactions is a tRNA-linked peptide generated by out-of-frame translation initiation from a cryptic Shine-Dalgarno sequence in the ubiquitin open reading frame. This peptidyl-tRNA does not interfere in the ubiquitination reactions. **(B)** FLAG-tagged CCT4, Ub-CCT4 and Ub(3A)-CCT4 were translated in RRL in the presence of 20 μM E1 inhibitor (TAK-243) and subjected to FLAG IP under native conditions. Input and IP samples were then analyzed by immunoblot. **(C)** ^{35}S-methionine-labelled Ub-GFP translated in the PURE system was incubated with E1, E2 (UBE2A), His-Ub (WT, K0 or the indicated K-to-R mutants), ATP and recombinant UBR4 and KCMF1 as indicated. The samples were then analyzed by autoradiography either directly (Input) or after a His-Ub PD under denaturing conditions. **(D)** ^{35}S-methionine-labelled Ub-CCT4 translated in the PURE system was incubated with E1, E2 (UBE2A), His-Ub (WT or K48R), ATP and recombinant UBR4 and KCMF1 as indicated. The samples were then analyzed by autoradiography either directly (Input) or after a His-Ub PD under denaturing conditions. **(E)** As in panel D except using Ub-PSMB4 as the substrate.
